# Supplementary material for: Data source and utilization of artificial intelligence technologies in vascular surgery—a scoping review
Source: Front Cardiovasc Med. 2025 May 7;12:1497822. doi: 10.3389/fcvm.2025.1497822 (PMC12093488; doi:10.3389/fcvm.2025.1497822)
Supplement: Supplementary file 1 [file Datasheet1.pdf]

Short overview of AI technologies

## Machine learning

Machine learning (ML) is based on probabilistic and statistical techniques forming complex systems that can iteratively learn from data (1). In general, the efficiency and the effectiveness of a machine learning solution depends on the characteristics of data and the performance of the learning algorithms. Therefore, selecting a proper learning algorithm that is suitable for the target application in a particular domain might be challenging. The reason is that the purpose of different learning algorithms is different, even the outcome of different learning algorithms in a similar category may vary depending on the data characteristics (2). The availability of the data is considered as a key to construct a machine learning model. There are generally three approaches that ML can utilise: supervised, unsupervised and reinforcement learning (Figure 1) (2,3). Supervised learning uses previously labelled data to learn its features, so it can perform on similar but unlabelled data; the most common supervised learning algorithms are classification and regression. Unsupervised learning analyses unlabelled data without the need for human interference and is widely used for extracting generative features, identifying meaningful trends and structures, groupings in results, and exploratory purposes; the commonplace unsupervised learning algorithms are clustering, density estimation, feature learning, dimensionality reduction and finding association rules. Hybridisation of these two methods is called a semi-supervised approach, that aims to provide a better outcome for the prediction than using the labelled data alone from the model; it is used in machine translation, labelling data and text classification. Reinforcement learning enables software to automatically evaluate the optimal behaviour in a particular context or environment to improve its efficiency; it is a powerful tool for training AI models that can help increase automation or optimise the operational efficiency of sophisticated systems (2,4).

Supplementary Figure 1. Machine learning techniques.

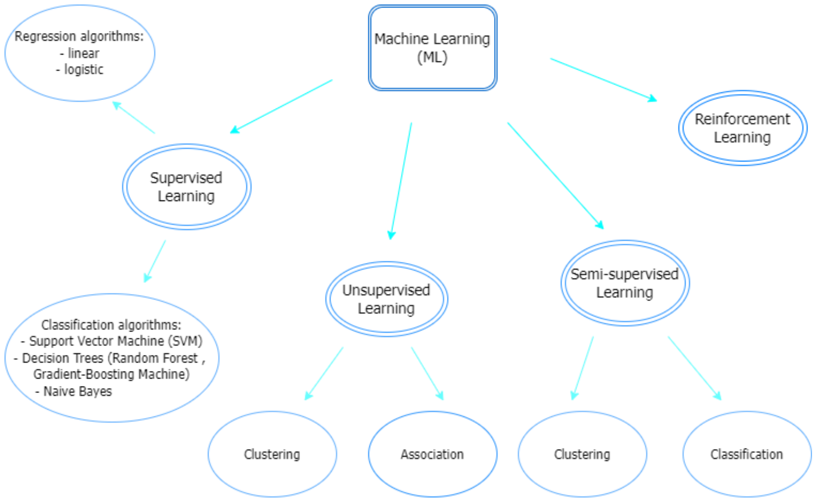


To solve a machine learning problem, the system is required in which a machine learning algorithm is only a part of it. The most important aspects of such a system are algorithm, and its choice is determined by the task at hand; training data (labelled and unlabelled); representation of data, which are expressed in terms of chosen features; a goal of the algorithm; and target, which essentially means what was learnt as well as the output of the algorithm (4). It is important to create, apart from training set, the validation and test sets, as the first is used for tuning the algorithm during the training session and the second for final evaluation and confirmation of the validity of the created algorithm (4).

## Deep Learning

Deep learning (DL), being a sub-field of ML, is a part of wider family of artificial neural networks (ANN) based machine learning approaches with representation learning. DL provides a computational architecture by combining several processing layers, such as input, hidden and output layers to learn from data. The main advantage of a DL over traditional ML is its better performance in learning from large data sets (2,4,5). The most common deep learning networks are Multilayer Perceptron (MLP), Convolutional Neural Network (CNN), and Recurrent Neural Network (RNN). MLP is a class of ANN, and its models are the most basic deep neural network. CNN is another class of DL models composed of different layers for processing data that has a grid pattern, such as images (Figure 2) (5). RNN is another class of DL models that is mainly applied in the field of speech processing and natural language processing (NLP) contexts. Unlike conventional networks, RNN uses sequential data in the network and includes less feature compatibility when compared to CNN (6).

Supplementary Figure 2. Example of a CNN architecture.

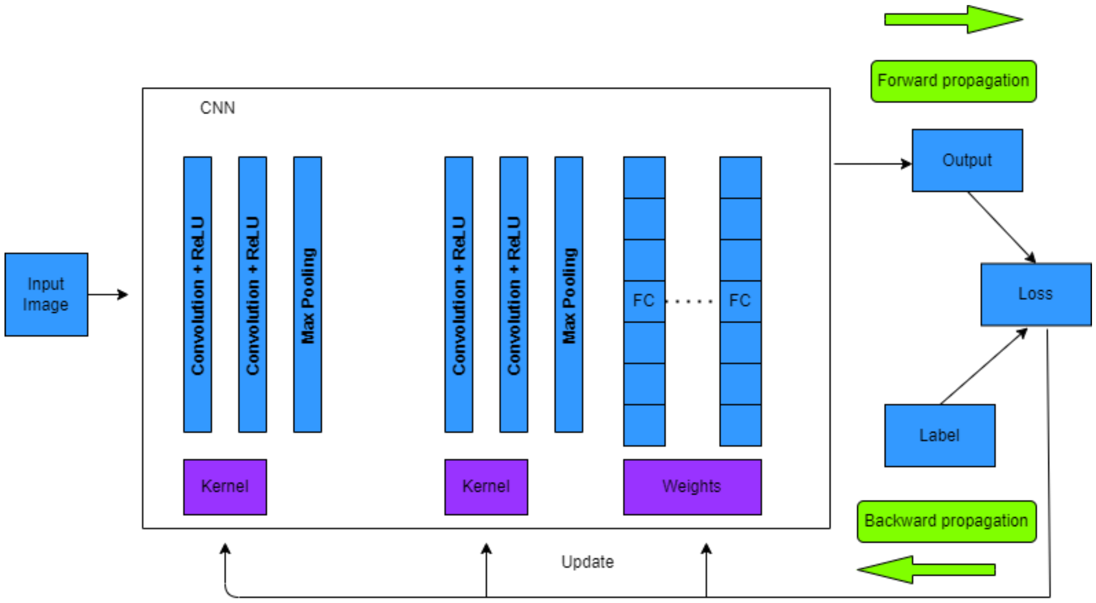


Deep Learning approaches in their concepts are similar to those of machine learning, and include: supervised learning, which is employed by RNNS, CNNs, and deep neural networks (DNNs); semi-supervised learning, which is employed by generative adversarial networks (GANs), deep reinforcement learning (DRL), and occasionally by RNNs; and unsupervised learning, which is used by generative networks, dimensionality reduction and clustering, such as auto-encoders, GANs, and RNNs (6).

## Natural Language Processing

Natural language processing (NLP) refers to the machine's ability to identify, process, understand and/or generate information in written and spoken human communications (7). Meaning is a fundamental concept in Natural Language Processing (NLP), in the tasks of both Natural Language Understanding (NLU) and Natural Language Generation (NLG) (8). Human language is composed of four major building blocks: phonemes, morphemes and lexemes, syntax and context as presented in Figure 3, and semantics is critical to understanding data and analytics results (9).

Supplementary Figure 3. Building blocks of language and their applications.

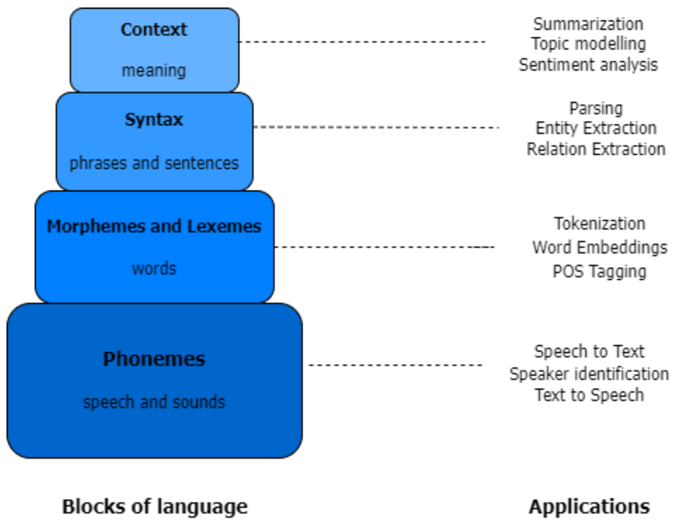


The process of language analysis is being perceived as being decomposable into a number of stages, mirroring the theoretical linguistic distinctions drawn between syntax, semantics and pragmatics. The sentences of a text are first analysed in terms of their syntax, which provides an order and structure that is more amenable to an analysis in terms of semantics, that provides a literal meaning. This is then followed by a stage of pragmatic analysis whereby the meaning of the utterance or text is determined (10). NLP applies computational (ML- or DL-based) or more basic statistical (heuristic-based) approaches to analyse speech and text and its tools have been used for information retrieval, classification, text extraction, text summarisation, question answering, and text generation utilising linguistics-focused or statistics-focused approaches, or a combination of these two (11,12). Heuristics-based method has been used in early attempts at designing NLP systems based on building rules for solving a problem at hand. Such systems require that the developers have some expertise in the domain to formulate rules that could be incorporated into a program, dictionaries and thesauruses and more elaborate knowledge bases having been built to aid NLP in general and rule-based NLP in particular, such as lexicon-based sentiment analysis or regular expressions (regex) (9). Machine learning-based methods to NLP can utilise supervised, semi-supervised and unsupervised techniques that are applied to textual data (9). Supervised machine learning techniques, such as classification or regression methods are heavily used for various NLP tasks. Deep learning-based approaches to NLP have been increasingly utilised in the language processing tasks. One of the neural networks employed in sequential language processing is recurrent neural network (RNN). Convolutional neural network (CNN) has been successfully employed in NLP, especially in the text-classification tasks. The latest

entry in the league of deep learning models for NLP are transformers, which are designed to model the textual context but not in a sequential manner and are used for transfer learning, where the knowledge gained while solving one problem is applied to a different, but related problem (6,9).

## Reference


1. Javidan AP, Li A, Lee MH, Forbes TL, Naji F. A Systematic Review and Bibliometric Analysis of Applications of Artificial Intelligence and Machine Learning in Vascular Surgery. Ann Vasc Surg [Internet]. 2022;85:395–405. Available from: https://doi.org/10.1016/j.avsg.2022.03.019

2. Sarker IH. Machine Learning: Algorithms, Real-World Applications and Research Directions. SN Comput Sci [Internet]. 2021;2(3):1–21. Available from: https://doi.org/10.1007/s42979-021-00592-x

3. Vasilev I, Slater D, Spacagna G, Roelants P, Zocca V. Python Deep Learning [Internet]. 2019. 383 p. Available from: https://books.google.com/books?hl=en&lr=&id=2UEwDwAAQBAJ&oi=fnd&pg=PP1&dq=data+science+and+python&ots=RoIMHjWy6l&sig=Xrnvhn834NPKtUb0rQxWq10V_vo

4. Zocca V, Spacagna G, Slater D, Roelants P. Python Deep Learning. Packt Publishing; 2017.

5. Yamashita R, Nishio M, Do RKG, Togashi K. Convolutional Neural Networks: An Overview and Its Applications in Pattern Recognition. Insights Imaging. 2018;9:611–29.

6. Alzubaidi L, Zhang J, Humaidi AJ, Al-Dujaili A, Duan Y, Al-Shamma O, et al. Review of deep learning: concepts, CNN architectures, challenges, applications, future directions [Internet]. Vol. 8, Journal of Big Data. Springer International Publishing; 2021. Available from: https://doi.org/10.1186/s40537-021-00444-8

7. Samoli S, Lopez Cobo M, De Prato G, Martinez-Plumed F, Delipetrev B. AI Watch - Defining Artificial Intelligence. Towards an operational definition and taxonomy of artificial intelligence [Internet]. Joint Research Centre (European Commission). 2020. 1–90 p. Available from: https://ec.europa.eu/jrc

8. Bender EM, Lascarides A. Linguistic fundamentals for natural language processing [Internet]. 2013. 166 p. Available from: http://cds.cern.ch/record/1604092

9. Vajjala S, Majmuder B, Gupta A, Surana H. Practical Natural Language Processing_ A Comprehensive Guide to Building Real-world NLPV Systems. O'Reilly Media, Inc. 2020;325.

10. Indurkhya N, Damerau FJ. Handbook of Natural Language Processing. Chapman Hall/CRC, Mach Learn Pattern Recognit Ser. 2010;

11. Linna N, Kahn CE. Applications of natural language processing in radiology: A systematic review. Int J Med Inform [Internet]. 2022;163(March):104779. Available from: https://doi.org/10.1016/j.ijmedinf.2022.104779

12. Wong A, Plasek JM, Montecalvo SP, Zhou L. Natural Language Processing and Its Implications for the Future of Medication Safety: A Narrative Review of Recent Advances and Challenges. Pharmacotherapy. 2018;38(8):822–41.
